# Draft genome sequences of *Burkholderia cepacia* complex causing nosocomial bacteremia in neonates admitted to the Manhiça District Hospital, Southern Mozambique

**DOI:** 10.1128/mra.00389-26

**Published:** 2026-06-03

**Authors:** Augusto Messa, Marcelino Garrine, Avertino Benedito, Janeta Machai, Sérgio Massora, Tacilta Nhampossa, Inácio Mandomando

**Affiliations:** 1 Centro de Investigação em Saúde de Manhiça (CISM), Maputo, Mozambique; 2 ISGlobal 310844, Barcelona, Spain; 3 Facultat de Medicina i Ciències de la Salut, Universitat de Barcelona (UB) 16724, Barcelona, Spain; 4 Global Health and Tropical Medicine, GHTM, Associate Laboratory in Translation and Innovation Towards Global Health, LA-REAL, Instituto de Higiene e Medicina Tropical, IHMT, Universidade NOVA de Lisboa 50106, Lisboa, Portugal; 5 Instituto Nacional de Saúde (INS) 586683, Maputo, Mozambique; University of Manitoba, Winnipeg, Canada

**Keywords:** *Burkholderia cepacia* complex, bacteremia, nosocomial infection, whole-genome sequencing (WGS), Mozambique, antimicrobial resistance (AMR)

## Abstract

*Burkholderia cepacia* complex (Bcc) are opportunistic and environmental pathogens associated with nosocomial bacteremia, yet data from low-income countries are scarce. Here, we report the draft whole-genome sequences of Bcc from blood cultures of admitted patients (*n* = 16) and the hospital environment (*n* = 5) at the Manhiça District Hospital.

## ANNOUNCEMENT

*Burkholderia cepacia* complex (Bcc) is a group of environmental and opportunistic non-spore-forming gram-negative bacilli comprising more than 20 species, commonly shown to possess resistance to multiple classes of antimicrobials (1–3). *Burkholderia cenocepacia*, *Burkholderia multivorans*, *Burkholderia dolosa*, and *Burkholderia contaminans* frequently cause nosocomial infections in neonates, immunocompromised individuals, and cystic fibrosis patients, resulting in bacteremia from indwelling catheters, urinary tract infections, septic arthritis, peritonitis, and respiratory tract infections (4–8). Sources for nosocomial infections include taps, medications, suction equipment, and water (7–9). There is scarce information on Bcc in Sub-Saharan Africa. Herein, we announce draft whole-genome sequences of eight *B. cenocepacia*, seven *B. contaminans*, and one *Burkholderia gladioli* isolated from the blood cultures of admitted patients and five *B. contaminans* from the hospital environment in the Manhiça District Hospital (MDH), Southern Mozambique, between 2020 and 2023.

The draft Bcc genomes described in this report were obtained following the whole-genome sequencing of strains from blood samples collected upon admission of children <2 years or children aged 2–14 years, and adults with axillary temperature ≥39°C or with signs of severe illness within the invasive bacterial disease surveillance at the MDH as previously described (10). We also describe the genomes of strains isolated from hospital environment samples (swabs of surfaces and instruments in the pediatric ward) collected in a nosocomial outbreak investigation. The isolates were typed by multilocus sequence typing (MLST), using the Bcc scheme with the *recA* gene used for species identification (11, 12). Pure fresh colonies subcultured on Tryptone Soya Agar and incubated at 37°C for 18–24 h were used for genomic DNA extraction using the QIAamp DNA Mini Kit (Qiagen, Germany) and quantified on a Qubit fluorometer using a High Sensitivity dsDNA Assay Kit (Thermo Fisher Scientific, USA). Genomic libraries were prepared using the Nextera XT Library preparation protocol (Illumina) for obtaining 150-bp paired-end reads, followed by sequencing with Illumina MiSeq (*B. cenocepacia*) or HiSeq (*B. contaminans* and *B. gladioli*) instruments. All commercial kits were used following the vendor’s protocols without modifications.

Raw reads were processed using fastp v.0.26.0 (13) and *de novo* assemblies generated using the shovill v.1.1.0 (https://github.com/tseemann/shovill) pipeline that uses SPAdes v3.15.5 (14) and then evaluated with QUAST v.5.2.0 (15). Genome completeness and contamination were assessed through CheckM v.1.2.2 (16) and MLST confirmed *in silico* using mlst (https://github.com/tseemann/mlst). Upon submission to the National Center for Biotechnology Information (NCBI), draft genomes were annotated using the Prokaryotic Genome Annotation Pipeline v.6.10 (17). The presence of antimicrobial resistance (AMR) genes was analyzed through ABRicate v1.0.1 (https://github.com/tseemann/abricate) using ncbi, resfinder, and plasmidfinder databases, in addition to NCBI AMRFinder Plus v3.12.8 (18) and ResFinder v4.4.3 (19) command line tools. All software tools were used with default parameters.

Table 1 presents key genome statistics and AMR determinants identified *in silico*. These genomes provide a valuable resource for comparative genomics and phylogenetic studies of nosocomial infections by Bcc in Mozambique and Sub-Saharan Africa, especially with description of novel STs.

**TABLE 1:** NCBI accession numbers, assembly metrics, and detected antimicrobial resistance (AMR) genes for each isolate analyzed in this study

Isolate (source sample type)	SRA accession (BioSample); genome assembly accession^*a*^	Species	Sequence type	Nr of reads (Nr. of bases)	Genome size, bp (GC, %)	Genome coverage^*d*^	Number of contigs (>500 bp)	N50, bp (L50)	Completeness, % (contamination, %)^*f*^	Nr of genes (coding)	Antimicrobial resistance genes
1785371_2 (blood)	SRR28968521 (SAMN41266729); JBWJFE000000000	*B. cenocepacia*	1890 (novel^*b*^)	5294662 (693751230)	8009678 (67)	91×	554 (473)	29699 (78)	99.26 (2.16)	7597 (7413)	*bla*_OXA-1043_, *bla*_PEN-B1_, *dfrA5*, *sul1*, *ere(A*), *tet(64*), *qacE*Δ1
1785313_2 (blood)	SRR28968535 (SAMN41266726); JBWJFD000000000	*B. cenocepacia*	1890 (novel^*b*^)	4612626 (609612607)	8003196 (67)	81×	580 (483)	29671 (80)	99.57 (1.58)	7598 (7412)	*bla*_OXA-1043_, *bla*_PEN-B1_, *dfrA5*, *sul1*, *ere(A*), *tet(64*), *qacE*Δ1
1785322_4 (blood)	SRR28968534 (SAMN41266727); JBWJFC000000000	*B. cenocepacia*	1890 (novel^*b*^)	4226696 (559149139)	7983685 (66.97)	76×	788 (689)	22158 (102)	99.58 (2.38)	7719 (7521)	*bla*_OXA-1043_, *bla*_PEN-B1_, *dfrA5*, *sul1*, *ere(A*), *tet(64*), *qacE*Δ1
1808361_3 (blood)	SRR28968519 (SAMN41266731); JBWJFB000000000	*B. cenocepacia*	1890 (novel^*b*^)	2873396 (376978074)	7996005 (66.98)	55×	796 (691)	20958 (108)	99.41 (1.99)	7709 (7510)	*bla*_OXA-1043_, *bla*_PEN-B1_, *dfrA5*, *sul1*, *ere(A*), *tet(64*), *qacE*Δ1
1785344_6 (blood)	SRR28968523 (SAMN41266728); JBWJFA000000000	*B. cenocepacia*	1890 (novel^*b*^)	3568564 (469419805)	7999258 (66.99)	64×	649 (537)	25888 (89)	99.26 (1.58)	7609 (7419)	*bla*_OXA-1043_, *bla*_PEN-B1_, *dfrA5*, *sul1*, *ere(A*), *tet(64*), *qacE*
1785380_4 (blood)	SRR28968520 (SAMN41266730); JBWJEZ000000000	*B. cenocepacia*	1890 (novel^*b*^)	4733844 (614457751)	8021377 (67.02)	80×	429 (347)	44166 (60)	99.78 (1.58)	7536 (7369)	*bla*_OXA-1043_, *bla*_PEN-B1_, *dfrA5*, *sul1*, *ere(A*), *tet(64*), *qacE*
1830240_0 (blood)	SRR28968517 (SAMN41266733); JBWJEY000000000	*B. cenocepacia*	1890 (novel^*b*^)	2717916 (353238156)	7856499 (66.83)	57×	1513 (1393)	9151 (222)	97.52 (2.79)	8083 (7857)	*bla*_OXA-1043_, *bla*_PEN-B1_, *dfrA5*, *sul1*, *ere(A*), *tet(64*), *qacE*
1808391_0 (blood)	SRR28968518 (SAMN41266732); JBWJEX000000000	*B. cenocepacia*	1890 (novel^*b*^)	3458624 (451801050)	7917807 (66.98)	66×	1346 (1236)	10680 (186)	97.95 (2.59)	8030 (7807)	*bla*_OXA-1043_, *bla*_PEN-B1_, *dfrA5*, *sul1*, *ere(A*), *tet(64*), *qacE*
2086484_2 (blood)	SRR28968530 (SAMN41266739); JBWJEW000000000	*B. contaminans*	102	20962280 (3.100253 G)	8917882 (66.12)	348×^*e*^	160 (101)	237857 (9)	100 (0.42)	8191 (7794)	*bla*_PEN-BCC_, *tet(64*)
2086461_3 (blood)	SRR28968531 (SAMN41266738); JBWJEV000000000	*B. contaminans*	102	20132794 (2.943245 G)	8924256 (66.12)	330×^*e*^	143 (83)	270770 (9)	100 (0.42)	8181 (7980)	*bla*_PEN-BCC_, *tet(64*)
2085810_0 (blood)	SRR28968532 (SAMN41266737); JBWJEU000000000	*B. contaminans*	102	20971638 (3.102135 G)	8924884 (66.12)	348×^*e*^	150 (83)	280843 (8)	100 (0.42)	8185 (7982)	*bla*_PEN-BCC_, *tet(64*)
2086498_9 (blood)	SRR28968529 (SAMN41266740); JBWJET000000000	*B. contaminans*	102	21229698 (3.144416 G)	8925105 (66.12)	353×^*e*^	144 (85)	270770 (9)	100 (0.42)	8180 (7978)	*bla*_PEN-BCC_, *tet(64*)
2085750_9 (blood)	SRR28968533 (SAMN41266736); JBWJES000000000	*B. contaminans*	102	20243470 (2.982369 G)	8925635 (66.12)	334×^*e*^	135 (82)	280843 (10)	100 (0.42)	8175 (7975)	*bla*_PEN-BCC_, *tet(64*)
2040454_3 (blood)	SRR28968515 (SAMN41266735); JBWJER000000000	*B. contaminans*	102	19335790 (2.846366 G)	8925287 (66.12)	319×^*e*^	154 (84)	280843 (8)	100 (0.42)	8184 (7983)	*bla*_PEN-BCC_, *tet(64*)
2040452_9 (blood)	SRR28968516 (SAMN41266734); JBWJEQ000000000	*B. contaminans*	102	20079232 (2.956453 G)	8925586 (66.12)	332×^*e*^	144 (81)	296109 (8)	100 (0.42)	8176 (7975)	*bla*_PEN-BCC_, *tet(64*)
Z-28 (saline solution)	SRR28968526 (SAMN41266743); JBWJEP000000000	*B. contaminans*	102	19417104 (2.870609 G)	8928984 (66.12)	322×^*e*^	180 (89)	223985 (10)	100 (0.42)	8191 (7994)	*bla*_PEN-BCC_, *tet(64*)
Z-18 (10% dextrose solution)	SRR28968528 (SAMN41266741); JBWJEO000000000	*B. contaminans*	102	19263906 (2.836790 G)	8924982 (66.12)	318×^*e*^	127 (76)	280843 (8)	100 (0.42)	8172 (7971)	*bla*_PEN-BCC_, *tet(64*)
Z-19 (syringe for solution preparation)	SRR28968527 (SAMN41266742); JBWJEN000000000	*B. contaminans*	102	18960470 (2.804278 G)	8924242 (66.12)	315×^*e*^	151 (80)	390653 (7)	100 (0.42)	8184 (7986)	*bla*_PEN-BCC_, *tet(64*)
Z-57 (10% dextrose solution)	SRR28968525 (SAMN41266744); JBWJEM000000000	*B. contaminans*	102	19867398 (2.945530 G)	8929891 (66.12)	331×^*e*^	113 (73)	433656 (7)	100 (0.42)	8202 (7988)	*bla*_PEN-BCC_, *tet(64*)
Z-58 (glucose solution)	SRR28968524 (SAMN41266745); JBWJEL000000000	*B. contaminans*	102	19654256 (2.895199 G)	8925695 (66.12)	324×^*e*^	136 (79)	270770 (9)	100 (0.42)	8177 (7977)	*bla*_PEN-BCC_, *tet(64*)
2085882_7 (blood)	SRR28968522 (SAMN41266746); JBWJEK000000000	*B. gladioli*	2349 (novel^*c*^)	19283476 (2.851013 G)	8614985 (68.14)	332×^*e*^	115 (58)	388591 (7)	100 (1,66)	7612 (7454)	*bla*_PEN-N_

^*a*^ Draft genome assemblies submitted were trimmed to remove adapter sequences and other potential contaminants; only contigs >200 bp were included in the sequences submitted to NCBI Genomes database.

^*b*^ ST1890, a single locus variant of ST817.

^*c*^ ST2349, a double locus variant of ST1622.

^*d*^ Estimated by shovill v.1.1.0.

^*e*^ Downsampled to ~150× for genome assembly with shovill.

^*f*^ Estimated by CheckM v.1.2.2.

## Data Availability

The Whole Genome Shotgun project has been deposited at DDBJ/ENA/GenBank under BioProject accession PRJNA1109395. Sequence Read Archive, BioSample, and Genome assemblies accessions are listed and hyperlinked in Table 1 for each genome.
